# Education‐based differences in alcohol health literacy in Germany

**DOI:** 10.1111/dar.13985

**Published:** 2024-12-03

**Authors:** Carolin Kilian, Moritz Liebig, Jakob Manthey

**Affiliations:** ^1^ Center for Interdisciplinary Addiction Research, Department of Psychiatry and Psychotherapy University Medical Center Hamburg‐Eppendorf Hamburg Germany; ^2^ Institute for Mental Health Policy Research Centre for Addiction and Mental Health Toronto Canada; ^3^ Institute for Interdisciplinary Addiction and Drug Research Hamburg Germany; ^4^ Department of Social Sciences, Faculty of Business, Economics and Social Sciences University of Hamburg Hamburg Germany; ^5^ Department of Psychiatry, Medical Faculty University of Leipzig Leipzig Germany

**Keywords:** alcohol use, health education, health literacy, substance use

## Abstract

**Introduction:**

Alcohol health literacy is critical for informed consumer decision making but has yet received limited attention in public health research. We therefore seek to measure alcohol health literacy and its educational distribution in Germany.

**Methods:**

In this cross‐sectional study, we developed and applied a brief nine‐item questionnaire on alcohol health literacy in an adult convenience sample (*n* = 391; February to April 2023). The association of educational attainment with ‘insufficient’ alcohol health literacy was tested in adjusted logistic regression models.

**Results:**

Insufficient alcohol health literacy was recorded in 47.8% of men and 41.1% of women in our sample. While most respondents correctly identified common misconceptions and wrong beliefs about alcohol and were able to specify low‐risk drinking limits for women and women during pregnancy, only few correctly identified all alcohol‐related health conditions, especially respiratory and infectious diseases. Respondents with low education were 1.35 (risk ratio [RR], 95% confidence interval 1.09–1.50, *p* = 0.014) times more likely to have been classified as having insufficient alcohol health literacy than high‐educated respondents. There was no statistically significant difference between respondents with medium versus high education (RR = 1.22, 95% confidence interval 0.99–1.43, *p* = 0.060).

**Discussion and Conclusions:**

Educational gaps in alcohol health literacy question a policy rationale that is fundamentally based on the premise of informed consumer choice. Strategies to raise alcohol health literacy must ensure that they reach all population groups, for instance, by providing health warning labels on alcohol containers.

## 1 INTRODUCTION

Alcohol use is causally linked to more than 200 diseases and injuries [1], rendering it a major contributor to the health burden in Europe. In numbers, more than 583,000 people died due to an alcohol‐attributable cause in 2019 in the World Health Organization European Region, equivalent to 6% (95% confidence interval [CI] 5.4–7.1%) of all deaths according to the Global Burden of Disease study [2]. The three major disease categories are digestive diseases, neoplasms and cardiovascular diseases with 17.7 (95% CI 14.5–20.5), 14.4 (95% CI 12.9–15.9) and 12.2 (95% CI 5.6–19.1) deaths per 100,000. Despite ample epidemiological evidence demonstrating alcohol use as a major preventable risk factor for these diseases [1], there is a concerning lack of health literacy on these health risks in the European population. According to a recent online survey in 14 European countries, the knowledge about alcohol's causal role in the development of a range of diseases reached 90% for liver disease but dropped to 53% for cancers and only 11% for respiratory diseases (e.g., tuberculosis) [3].

Knowledge about the potential health hazards of a health‐related behaviour such as alcohol use is often discussed as a critical determinant to practicing this behaviour. However, just knowing about health risks appear to be insufficient to lead to behavioural change [4], especially for behaviours such as drinking alcohol, which is deeply rooted socially and culturally in many countries [5, 6]. The concept of health literacy acknowledges the complexity of making health‐related decisions [7] and involves multiple components: (i) knowledge and understanding (e.g., alcohol content in beverages, health risks); (ii) skills (e.g., access health‐related information); (iii) critical thinking (e.g., appraise marketing messages); and (iv) system competence (e.g., navigate in health systems; [8]). If alcohol health literacy is perceived in its full complexity, including higher‐level changes to the health care system and environment, for example, by reducing the affordability and availability of alcoholic beverages, it is very likely to lower population‐level alcohol consumption. In an expert panel study, education and information measures targeting the individual were rated most effective to increase alcohol health literacy, while being least effective to leading to an actual decline in alcohol use, for which alcohol control policies were rated the highest [9].

As most European countries follow a narrative of ‘responsible alcohol consumption’ characterised by limited regulations of alcoholic products [10, 11], high levels of alcohol health literacy are vital to public health. In fact, this liberal approach presupposes that all consumers can make informed decision by being able to access, understand and apply the relevant information to their day‐to‐day life in order to achieve desirable public health outcomes (e.g., reduced alcohol‐related health burden) [8]. However, this appears not to be the case as knowledge on alcohol's causal role in the development of different diseases were found to be higher in people with tertiary education compared to those with primary or secondary education in a sample of Europeans from 14 countries [3]. Such a correlation between years of education and health literacy has also been reported in the reach of German alcohol prevention campaigns [12] and on general health literacy (e.g., [13]), suggesting that the current narrative of ‘responsible alcohol consumption’ may penalise people with lower levels of education (see [14]).

Against this backdrop, we aim: (i) to capture the level of alcohol health literacy in the general adult population in Germany; as well as (ii) to investigate education‐based differences therein. We conduct our research in Germany that stands out for very high levels of alcohol use (alcohol sales in 2019: 10.6 L of pure alcohol, per capita [15]) and very liberal alcohol regulations [16]. While alcohol consumption and harms have declined slightly in the past decade in Germany, the alcohol‐attributable burden remains high [17]. Currently, there is no data available on how alcohol health literacy is distributed in Germany. We hypothesise that alcohol health literacy is significantly lower in people with low or medium levels of education compared to those with high education.

## 2 METHODS

### 2.1 Study design and data collection

We conducted a cross‐sectional survey of a convenience sample drawn from the general adult (18+ years) population in Germany. The survey was developed for the purpose of this study and comprised 29 items, covering questions on: (i) alcohol consumption (based on the AUDIT‐C [18], Lübecker translation); (ii) alcohol health literacy; (iii) general health literacy (HLS‐EU‐Q6 [19]); and (iv) socio‐demographics (for details, see below).

The questionnaire was implemented online via LimeSurvey [20] and participation was possible between 27 February 2023 and 11 April 2023. Respondents had to be at least 18 years of age to be eligible for participation. Potential respondents were recruited by using the personal networks of the study authors, the professional network of the Center for Interdisciplinary Addiction Research (University Medical Center Hamburg‐Eppendorf, Hamburg) and paid advertisements (for 500 clicks) on Facebook. This study's sample was limited to respondents who completed the survey (i.e., provided any answers to all items), which resulted in a study sample of *n* = 611 respondents.

This study was approved by the local psychological ethics committee of the Center for Psychosocial Medicine, University Medical Center Hamburg‐Eppendorf, Hamburg (2023/02/08).

### 2.2 Measures

#### 2.2.1 Dependent variable

Alcohol health literacy was measured by a composite score that was based on nine items (see Table S1, Supporting Information). The questionnaire was developed under consideration of available topic‐related surveys (e.g., the World Health Organization survey on alcohol labelling [3]) and used a similar item scale ranging from 1 (lowest) to 4 (highest) as the HLS‐EU‐Q6 questionnaire [19] to enable comparisons. Participants were asked about their knowledge regarding alcohol‐related diseases (one item), typical misconceptions and wrong beliefs regarding alcohol (four items), German alcohol drinking recommendations (as of January 2023 [21]; three items), and their ability to access and understand information on alcohol‐related health risks (one item). One additional item inquired whether respondents feel well‐informed about alcohol‐related risks, which was, however, not included in the alcohol health literacy score as it does not measure alcohol knowledge (item 2.7, see Table S1).

The composite score was calculated as the sum of all nine items divided by the number of items and ranged between 1 (lowest) and 4 (highest). Each item had an equal weight in the composite score, though the first item on alcohol‐related diseases covered nine different health conditions and respondents had to identify all nine to receive the highest score (i.e., four points; three points for 8 conditions, two points for 6–7 conditions, one point for 5 or less conditions). The composite score was dichotomised to differentiate ‘sufficient’ (scores >3) from ‘insufficient’ (scores ≤3) alcohol health literacy, that is, ‘sufficient’ alcohol health literacy could be achieved by giving correct answers (four points) to most questions. Cut‐offs were adopted from previous research applying the HLS‐EU in Germany [19]. While the original HLS‐EU differentiates between three levels of health literacy (sufficient, problematic, inadequate), we grouped the two lower levels (problematic, inadequate) together to reflect ‘insufficient’ alcohol health literacy, given that only very few respondents (4%) had scores below or equal to two.

#### 2.2.2 Independent variable

Educational attainment was recorded in accordance to the International Standard Classification of Education (ISCED 11) and grouped into low, medium, and high based on the highest level of education completed [22]. In cases where respondents indicated to have not yet completed their education, we asked to indicate their anticipated level of education.

#### 2.2.3 Covariates

Respondents were asked about their gender (men, women, ‘other’) and age (continuous). The age variable was grouped into three broad age groups (18–34, 35–49, 50+ years [highest recorded age was 80 years]). Alcohol use based on the Alcohol Use Disorders Identification Test‐Consumption (AUDIT‐C) sum score (range: 0–12) was grouped into current non‐drinker (AUDIT‐C score = 0), low‐risk (AUDIT‐C score ≥1 and <4 and <5 for women and men, respectively), and high‐risk alcohol use (AUDIT‐C score ≥4 and ≥5 for women and men, respectively) [23]. General health literacy based on the HLS‐EU‐Q6 [19] was classified into ‘inadequate’ (scores ≤2), ‘problematic’ (scores 2–3), and ‘sufficient’ (scores ≥3) health literacy in line with previous studies [19].

### 2.3 Statistical analysis

This study does not build upon a study protocol; thus, all findings should be considered exploratory. We first used descriptive statistics (proportions, means) to describe our sample by alcohol health literacy status and gender, using chi^2^ test statistics for statistical inference. Next, to test our hypothesis, we built logistic regression models with alcohol health literacy and educational attainment as dependent and independent variables, respectively. A first model was adjusted for gender and age group only. In a second and third model, we added the AUDIT‐C and general health literacy, respectively. As we expected the outcome of interest (insufficient alcohol health literacy) to be a non‐rare event (prevalence >10%), odds ratios were recalculated into risk ratios to ease interpretation [24]. Level of statistical significance was α ≤ 0.05. Data cleaning was undertaken in Stata SE 15.1 [25] and statistical analysis in R version 4.2.1 [26].

## 3 RESULTS

### 3.1 Alcohol health literacy in the study population

Six‐hundred and eleven individuals completed our online survey, of which 162 (26.5%) were excluded due to missing information on alcohol health literacy (i.e., response option ‘Not specified’). Another 8 (1.3%), 1 (0.1%), 2 (0.3%) and 44 (7.2%) observations were excluded due to missing information on educational attainment, gender, alcohol use and general health literacy, respectively. As only three respondents indicated ‘other’ gender, we restricted our analysis to men and women. The final sample with complete data on key variables comprised of *n* = 228 men and *n* = 163 women, with a mean age of 44.1 (SD = 17.8) and 45.7 (SD = 15.5) years, respectively.

In our sample, insufficient alcohol health literacy was recorded in 47.8% of men and 41.1% of women. Table 1 depicts the descriptive sample statistics by alcohol health literacy status and gender. The proportion of men and women with insufficient and sufficient alcohol health literacy did not statistically significantly differ regarding their age, education and general health literacy distributions. However, there were statistically significant more men with sufficient alcohol health literacy reporting to currently abstain from alcohol (*n* = 23, 19.3%) compared to men with insufficient alcohol health literacy (*n* = 8, 7.3%). Among women, 1 in 10 indicated to not drink alcohol (for comparison, men: 13.5%).

**TABLE 1:** Descriptive sample statistics by alcohol health literacy and gender.

	Men (*n* = 228)				Women (*n* = 163)			
	All men, *n* (%)	Insufficient AHL, *n* (%)	Sufficient AHL, *n* (%)	Chi^2^ test	All women, *n* (%)	Insufficient AHL, *n* (%)	Sufficient AHL, *n* (%)	Chi^2^ test
Age groups, years								
18–34	83 (36.4)	39 (47.0)	44 (53.0)	Chi^2^(2) = 0.24, *p* = 0.888	44 (27.0)	17 (38.6)	27 (61.4)	Chi^2^(2) = 0.19, *p* = 0.911
35–49	44 (19.3)	20 (45.5)	24 (54.5)		44 (27.0)	18 (40.9)	26 (59.1)	
50+	101 (44.3)	50 (49.5)	51 (50.5)		75 (46.0)	32 (42.7)	43 (57.3)	
Educational attainment								
Low	14 (6.1)	10 (71.4)	4 (28.6)	Chi^2^(2) = 4.43, *p* = 0.109	12 (7.4)	6 (50.0)	6 (50.0)	Chi^2^(2) = 1.84, *p* = 0.398
Middle	102 (44.7)	51 (50.0)	51 (50.0)		63 (38.7)	29 (46.0)	34 (54.0)	
High	112 (49.1)	48 (42.9)	64 (57.1)		88 (54.0)	32 (36.4)	56 (63.6)	
General health literacy								
Inadequate	44 (19.3)	24 (54.5)	20 (45.5)	Chi^2^(2) = 1.10, *p* = 0.576	22 (13.5)	10 (45.5)	12 (54.5)	Chi^2^(2) = 1.77, *p* = 0.414
Problematic	147 (64.5)	67 (45.6)	80 (54.4)		118 (72.4)	45 (38.1)	73 (61.9)	
Sufficient	37 (16.2)	18 (48.6)	19 (51.4)		23 (14.1)	12 (52.2)	11 (47.8)	
Alcohol use								
Non‐drinker	31 (13.6)	8 (25.8)	23 (74.2)	Chi^2^(2) = 6.96, *p* = 0.031	15 (9.2)	6 (40.0)	9 (60.0)	Chi^2^(2) = 1.24, *p* = 0.537
Low risk	80 (35.1)	41 (51.3)	39 (48.7)		72 (44.2)	33 (45.8)	39 (54.2)	
High risk	117 (51.3)	60 (51.3)	57 (48.7)		76 (46.6)	28 (36.8)	48 (63.2)	

Abbreviation: AHL, alcohol health literacy.

Most respondents (≥75%) indicated that liver diseases, injuries, fetal damage, mental health conditions, and heart diseases are causally linked to alcohol use (Figure S1). The alcohol‐cancer link was known by 57.7% (low education) to 83.0% (high education), while alcohol's causal contribution to dementia was reported by 50.0% (low education) to 66.0% (high education)—suggesting an educational gradient as hypothesised. The role of alcohol use in respiratory and infectious diseases was identified by less than a quarter of respondents across educational groups. Moreover, at least three out of four respondents stated that drinking wine would benefit their health and that wine would be generally better for their health than the same amount of pure alcohol in beer (Figure S2).

Respondents were further asked to indicate the limits of at‐risk alcohol use according to the German drinking guidelines as of early 2023 (Figure S3). Most respondents were able to report the relevant drinking limits (per day, men: 2 drinks, women: 1 drink, women during pregnancy: 0 drinks). However, 23.1% to 34.6% of respondents from the low‐education group indicated values above the recommended limits, while this was the case in 11.0% to 18.8% of those in the other education groups.

Lastly, we asked respondents how easily they understand information on alcohol‐related risks (Figure S4). Across education groups, the majority indicated that they consider it easy to very easy to understand health‐related information on alcohol risks (low education: 61.6%, high education: 76%).

### 3.2 Educational differences in alcohol health literacy

Table 2 presents the results from the logistic regression models. According the fully adjusted model, respondents with low education were about 1.35 times more likely to have an insufficient alcohol health literacy compared to those with high education. Moreover, current non‐drinkers were significantly less likely to have an insufficient alcohol health literacy than low‐risk alcohol users, while there was no statistically significant difference between high‐risk and low‐risk alcohol users. All other covariates (gender, age group, general health literacy) were not statistically significantly associated with the outcome.

**TABLE 2:** Logistic regression for insufficient alcohol health literacy.

	Model 1			Model 2			Model 3		
	RR	95% CI	*p*‐value	RR	95% CI	*p*‐value	RR	95% CI	*p*‐value
Educational attainment (ref. high)									
Middle	1.19	0.97–1.39	0.098	1.21	0.99–1.42	0.067	1.22	0.99–1.43	0.060
Low	**1.31**	**1.04–1.48**	**0.028**	**1.35**	**1.1–1.5**	**0.012**	**1.35**	**1.09–1.5**	**0.014**
Gender (ref. men)									
Women	0.84	0.63–1.07	0.174	0.8	0.59–1.04	0.104	0.82	0.6–1.06	0.133
Age groups (ref. 18–34), years									
35–49	1.12	0.80–1.45	0.474	1.16	0.83–1.5	0.346	1.17	0.83–1.5	0.341
50+	1.12	0.87–1.37	0.361	1.15	0.9–1.4	0.252	1.13	0.88–1.39	0.318
Alcohol use (ref. low risk)									
Non‐drinker				**0.46**	**0.24–0.82**	**0.009**	**0.45**	**0.23–0.81**	**0.007**
High risk				0.91	0.69–1.15	0.459	0.90	0.68–1.15	0.404
General health literacy (ref. sufficient)									
Problematic							0.82	0.54–1.15	0.279
Inadequate							1.02	0.68–1.35	0.922

*Note*: Sample size: *n* = 391. Bold values, *p* < 0.05.

Abbreviations: CI, confidence interval; Model 1, adjusted for gender and age group; Model 2, adjusted for gender, age group, and alcohol use; Model 3, adjusted for gender, age group, alcohol use, and general health literacy; RR, risk ratio.

Given the high degree of missingness in the outcome variable, we ran sensitivity analysis using a unique category for missing alcohol health literacy and fitted a multinomial logistic regression (alcohol health literacy: sufficient, insufficient, missing). Including respondents with missing data on alcohol health literacy, the analytical sample size was substantially larger (*n* = 508). The results are shown in Table S2 and suggest no statistically significant association of missing alcohol health literacy data and education. Women were about 1.4 times more likely to have missing data compared to men. This significant association, however, diminished when adjusting for alcohol use and general health literacy. In multinomial regression analyses, the statistically significant association between low education and insufficient alcohol health literacy was about risk ratio = 1.6 across models.

## 4 DISCUSSION

In our exploratory study, we investigated the distribution of alcohol health literacy in a convenience sample of adults residing in Germany. We applied a brief nine‐item questionnaire of alcohol health literacy assessing knowledge pertaining to alcohol risks and guidelines of high‐risk alcohol use, as well as the understanding of health information related to alcohol use. In our sample, more than 50% of respondents were classified as having sufficient alcohol health literacy, defined as having comprehensive knowledge about alcohol‐related health harms and drinking guidelines in addition to the ability to access and understand this information. High levels of alcohol health literacy were mainly driven by most respondents correctly identifying misconceptions and wrong beliefs about alcohol, as well as by being able to correctly specify the low risk drinking limits for women and women during pregnancy. As people with lower levels of education were more likely to be classified as having insufficient alcohol health literacy, we can confirm our hypothesis.

### 4.1 Limitations

Before interpreting the results further, we would like to highlight four important limitations of our study. First, we used a novel set of questions to measure alcohol health literacy, which had not been validated prior to or in the current study. This was necessary due to lack of an existing instrument in German language. Although the questionnaire was based on published surveys in the field, we cannot ascertain that it reliably and validly captures alcohol health literacy. Future studies may use our set of questions to develop a more comprehensive tool to capture alcohol health literacy that is systematically evaluated for reliability and validity. Second, we must acknowledge the limitations inherent in the nature of convenience samples. We do not expect the prevalence estimates to be generalisable to Germany at large. There is no reason to believe that the sample biases the association examined between educational attainment and alcohol health literacy, but we cannot rule out this possibility. Third, some respondents may have looked up answers to knowledge questions. This may bias our findings if this was more likely done by people within a specific education group. In anonymous online surveys, there is a low threshold for manipulating responses, but as there was no incentive to do so, we assume that few, if any, respondents engaged in this behaviour. Lastly, we only included *n* = 26 persons with low educational attainment, precluding gender‐stratified analysis and resulting in limited confidence for hypothesis testing.

### 4.2 Interpretation

Comparing our results to a recent cross‐country study [3], we find remarkable similarities. As in our study, findings from 19,000 persons residing in 14 European countries show that knowledge pertaining to health risks from using alcohol is highest for liver diseases, followed by heart diseases and cancer, and lowest for respiratory diseases. People with higher education consistently reported better knowledge in that European study, but in our study, we find a possible reverse link (i.e., better knowledge among lower educated) for mental health and respiratory as well as infectious diseases (see Figure S1). Future studies should follow up whether the educational gap in knowledge differs by health outcome.

Given the observed education‐based differences in alcohol health literacy, the question arises as to why these exist. Obviously, the knowledge about potential health risks is an important component of alcohol health literacy [8] that was assessed in our questionnaire and is directly linked to education. However, there are also structural issues that may contribute to lower health literacy in low‐educated individuals. For example, the federal prevention campaign ‘Alkohol—Kenn dein Limit’ (translation: ‘Alcohol—know your limit’; Bundeszentrale für gesundheitliche Aufklärung) was found to have a larger reach to adolescents and young adults with higher educational attainments [12], potentially contributing to the observed education‐based gap in alcohol health literacy. In Germany, there are only very few campaigns addressing adults and these campaigns are often invisible in the day‐to‐day life of the people [27]. A very simple measure to raise awareness of alcohol's health risks is the introduction of health warning labels on alcohol containers [28], which, however, is currently not part of the public discourse in Germany.

Overall, the education‐based differences in alcohol health literacy were small to moderate (risk ratio 1.3 to 1.6). However, these small gaps should be interpreted in light of other possible determinants of increased harm among disadvantaged populations [29]. There is heterogeneous data on the education gradient with respect to alcohol use from Germany. General population data suggest that people with lower educational attainment appear to be less likely to engage in alcohol use in general [30] and risky use patterns [31]. In other words, the exposure to alcohol generally and high levels of alcohol specifically is below‐average for lower educated adults in Germany. Currently, there is no data available on the education gradient of alcohol harms in Germany. As for treatment, it appears that people with hazardous drinking patterns are twice as likely to be given a brief advice for hazardous drinking if they have a low educational background [32].

## 5 CONCLUSION

This is the first study undertaking an in‐depth exploration of alcohol health literacy and its educational distribution in Germany. We found respondents with low educational attainment to be more likely to have insufficient alcohol health literacy compared to those with high education. This educational gap is concerning given worse alcohol‐related health outcomes in individuals with low socio‐economic status [29], as it potentially increases socioeconomic differences in alcohol‐attributable morbidity and mortality. Moreover, our findings call into question the premise of the German alcohol policy that keeps alcohol largely unregulated [16] and is based on the notion of informed consumer choice. However, given the observed systematic education‐based differences in alcohol health literacy, this premise appears to be no longer valid. To increase alcohol health literacy across all population groups, a comprehensive strategy is needed, including effective prevention programs in schools, easily accessible information on alcohol use, information provided in simple language and languages other than German, as well as health warning labels on alcohol containers [9].

## AUTHOR CONTRIBUTIONS

Conceptualisation: Carolin Kilian, Moritz Liebig, Jakob Manthey; Data curation: Moritz Liebig; Formal analysis: Carolin Kilian, Moritz Liebig; Methodology: Carolin Kilian, Moritz Liebig; Supervision: Jakob Manthey; Validation: Carolin Kilian, Moritz Liebig; Visualisation: Carolin Kilian; Writing—original draft: Carolin Kilian; and writing—review and editing: all authors.

## CONFLICT OF INTEREST STATEMENT

None.

## Supporting information


**Data S1:** Supporting information.

## Data Availability

The questionnaire (doi: 10.6084/m9.figshare.26030566.v1), data (doi: 10.6084/m9.figshare.26030590.v1), and statistical codes (doi: 10.6084/m9.figshare.26030608.v1) that support the findings and conclusions from this research are available at the Figshare repository.
