# Supplementary material for: Education‐based differences in alcohol health literacy in Germany
Source: Drug Alcohol Rev. 2024 Dec 3;44(2):555–62. doi: 10.1111/dar.13985 (PMC11814345; doi:10.1111/dar.13985)
Supplement: Supplementary file 1 — Data S1: Supporting information. [file DAR-44-555-s001.docx]

# Supplementary information

**Title:** Education-based differences in alcohol health literacy in Germany

**Authors:** Carolin Kilian, Moritz Liebig, Jakob Manthey

Table S1. Survey questions on alcohol health literacy in German and English with scoring system.

| **Item #** | **German (original)** | **English translation** | **Points in scoring system** |
| --- | --- | --- | --- |
| F2.1 | Kann Alkoholkonsum Krankheiten verursachen? Wenn ja, wählen Sie bitte alle Krankheiten aus, die Ihrer Meinung nach zutreffen.   1. Krebs 2. Psychische Erkrankungen (z.B. Depressionen) 3. Erkrankungen der Leber 4. Infektionskrankheiten 5. Herzkrankheiten 6. Verletzungen durch Kontrollverlust 7. Demenzerkrankungen 8. Schäden am ungeborenen Kind 9. Atemwegserkrankungen 10. Alkohol kann keine Krankheiten verursachen 11. Keine Angabe | Can alcohol consumption cause diseases? If yes, please select all diseases, that are fitting the criteria.   1. Cancers 2. Mental illness (e.g., depression) 3. Liver diseases 4. Infectious diseases 5. Heart diseases 6. Injuries due to loss of control 7. Dementia 8. Harm to the unborn child 9. Respiratory diseases 10. Alcohol cannot cause any diseases 11. Not specified | - 4 points for all conditions (1-9) - 3 points for eight conditions - 2 points for six to seven conditions - 1 point for five or less conditions - 1 point for indicating 10 (alcohol cannot cause any diseases) |
| F2.2 | Wie viele kleine Gläser Wein (0,125L) oder Bier (0,33L) kann eine schwangere Person bei einer Gelegenheit trinken, ohne das ungeborene Kind zu gefährden?  Antwort: ______________________   1. Keine Angabe | How many small glasses of wine (0,125L) or beer (0,33L) can a pregnant women drink on the same occasion without endangering the unborn child?  Response: ______________________   1. Not specified | - 4 points for 0 glasses - 1 point for any other response |
| F2.3 | Wenn ich mehr Alkohol konsumiere, besteht ein größeres Risiko für gesundheitliche Schäden.   1. Stimme überhaupt nicht zu 2. Stimme nicht zu 3. Stimme zu 4. Stimme voll zu 5. Keine Angabe | If I consume more alcohol, there is a greater risk of harm to my health.   1. Do not agree at all 2. Do not agree 3. Agree 4. Fully agree 5. Not specified | - 4 points for “fully agree” - 3 points for “agree” - 2 points for “do not agree” - 1 point for “do not agree at all” |
| F2.4 | Wenn ich regelmäßig Alkohol konsumiere, habe ich ein höheres Risiko an Krebs zu erkranken.   1. Stimme überhaupt nicht zu 2. Stimme nicht zu 3. Stimme zu 4. Stimme voll zu 5. Keine Angabe | If I consume alcohol on a regular basis, I am at greater risk of cancer.   1. Do not agree at all 2. Do not agree 3. Agree 4. Fully agree 5. Not specified | - 4 points for “fully agree” - 3 points for “agree” - 2 points for “do not agree” - 1 point for “do not agree at all” |
| F2.5 | Wenn ich ein Glas Wein pro Tag trinke, wirkt sich dies positiv auf meine Gesundheit aus.   1. Stimme überhaupt nicht zu 2. Stimme nicht zu 3. Stimme zu 4. Stimme voll zu 5. Keine Angabe | If I drink a glass of wine on a daily basis, it will be beneficial to my health.   1. Do not agree at all 2. Do not agree 3. Agree 4. Fully agree 5. Not specified | - 4 points for “do not agree at all” - 3 points for “do not agree” - 2 points for “agree” - 1 point for “fully agree” |
| F2.6 | Es ist besser für meine Gesundheit ein kleines Glas Wein zu trinken, als eine kleine Flasche Bier zu trinken.   1. Stimme überhaupt nicht zu 2. Stimme nicht zu 3. Stimme zu 4. Stimme voll zu 5. Keine Angabe | It is more beneficial to my health to drink a small glass of wine, instead of a small bottle of beer.   1. Do not agree at all 2. Do not agree 3. Agree 4. Fully agree 5. Not specified | - 4 points for “do not agree at all” - 3 points for “do not agree” - 2 points for “agree” - 1 point for “fully agree” |
| F2.7 | Ich fühle mich auf alkoholbedingte Risiken bezogen gut aufgeklärt.   1. Stimme überhaupt nicht zu 2. Stimme nicht zu 3. Stimme zu 4. Stimme voll zu 5. Keine Angabe | I am feeling well informed about alcohol-related risks.   1. Do not agree at all 2. Do not agree 3. Agree 4. Fully agree 5. Not specified | Not included in composite score |
| F2.8 | Informationen über alkoholbedingte Schäden können über viele Quellen erworben werden. Beispiel hierfür sind Gesundheitszentren, Zeitungen und Zeitschriften, Freunde und Familie, sowie das Internet. Haben Sie solche Informationen schon einmal erhalten?   1. Ja (continue with 2.8.2) 2. Nein 3. Keine Angabe | Information about the harm caused by alcohol can come from many sources. Examples are health centres, newspapers and magazines, friends and family, and the internet. Have you received such information?   1. Yes (continue with 2.8.2) 2. No 3. Not specified | Continue with 2.8.2 |
| F2.8.2 | Wie leicht waren diese Informationen für Sie zu verstehen?   1. Sehr einfach 2. Eher einfach 3. Eher schwierig 4. Sehr schwierig 5. Keine Angabe | How easy was it for you to understand this information?   1. Very easy 2. Rather easy 3. Rather difficult 4. Very difficult 5. Not specified | - 4 points for “very easy” - 3 points for “rather easy” - 2 points for “rather difficult” - 1 point for “very difficult” |
| F2.9 | Sowohl für Männer als auch für Frauen gibt es Empfehlungen, wie viel Alkohol maximal am Tag getrunken werden sollte, um die Schwelle für risikoreichen Konsum nicht zu übersteigen. Was ist Ihrer Meinung nach die Anzahl an kleinen Gläsern Bier (0,33L) oder kleinen Gläsern Wein (0,125L) pro Tag, die ein Mann zu sich nehmen kann, ohne diesen Grenzwert zu übersteigen?  Antwort: ______________________   1. Keine Angabe | There are recommendations for both men and women on how much alcohol should be consumed per day at most to avoid exceeding the threshold for risky consumption. In your opinion, what is the number of small glasses of beer (0.33L) or small glasses of wine (0.125L) per day that a man can drink without exceeding this limit?  Response: ______________________   1. Not specified | - 4 points for 2 glasses - 1 point for any other response |
| F2.10 | Was ist Ihrer Meinung die Anzahl an kleinen Gläsern Bier (0,33L) oder kleinen Gläsern Wein (0,125L) pro Tag, die eine Frau zu sich nehmen kann, ohne den Grenzwert für risikoreichen Alkoholkonsum zu übersteigen?  Antwort: ______________________   1. Keine Angabe | In your opinion, what is the number of small glasses of beer (0.33L) or small glasses of wine (0.125L) per day that a woman can drink without exceeding the threshold for risky alcohol consumption?  Response: ______________________   1. Not specified | - 4 points for 1 glass - 1 point for any other response |


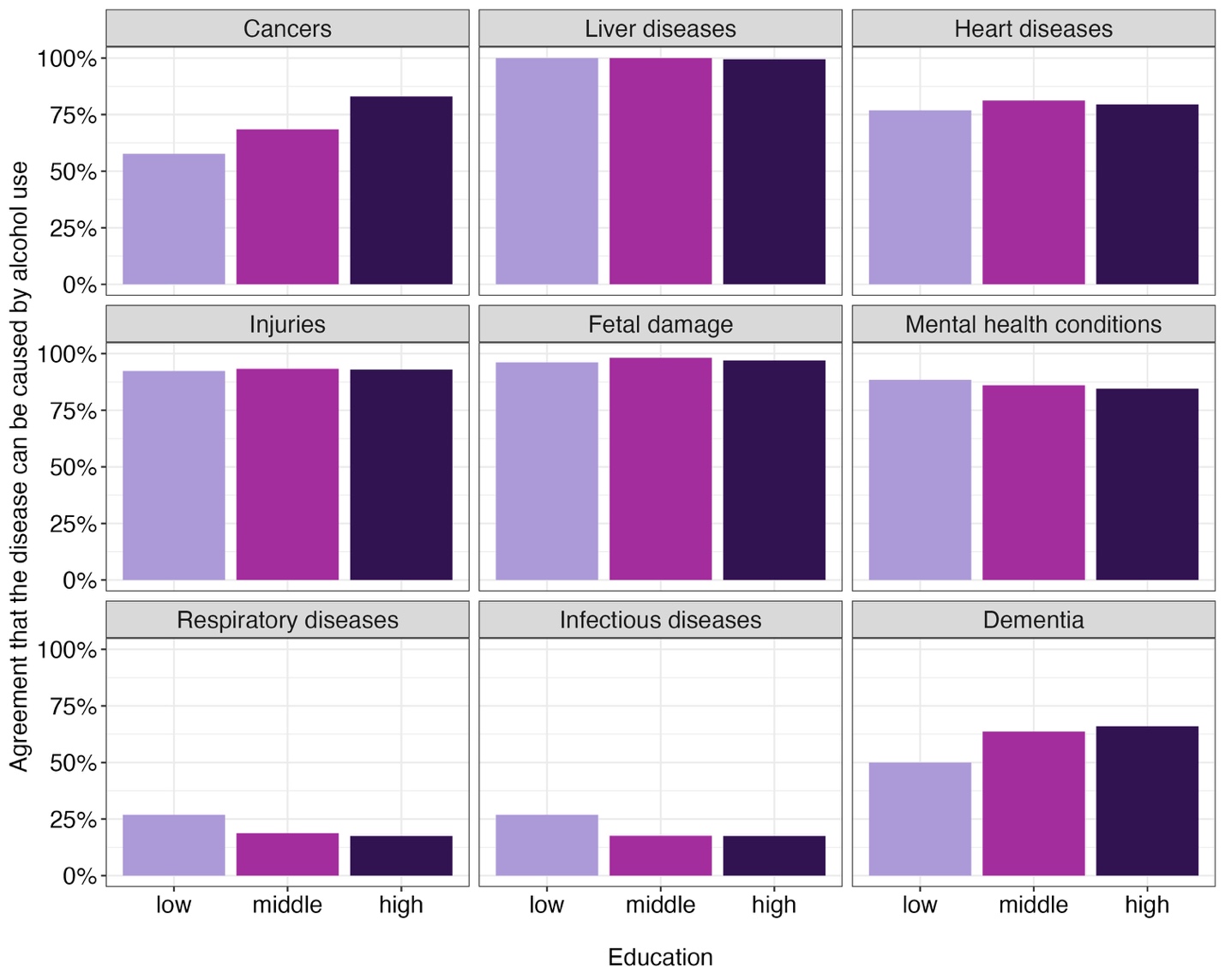


Figure S1. Percentage of respondents who agreed that different diseases are causally linked to alcohol use, by education group (alcohol health literacy item #1).


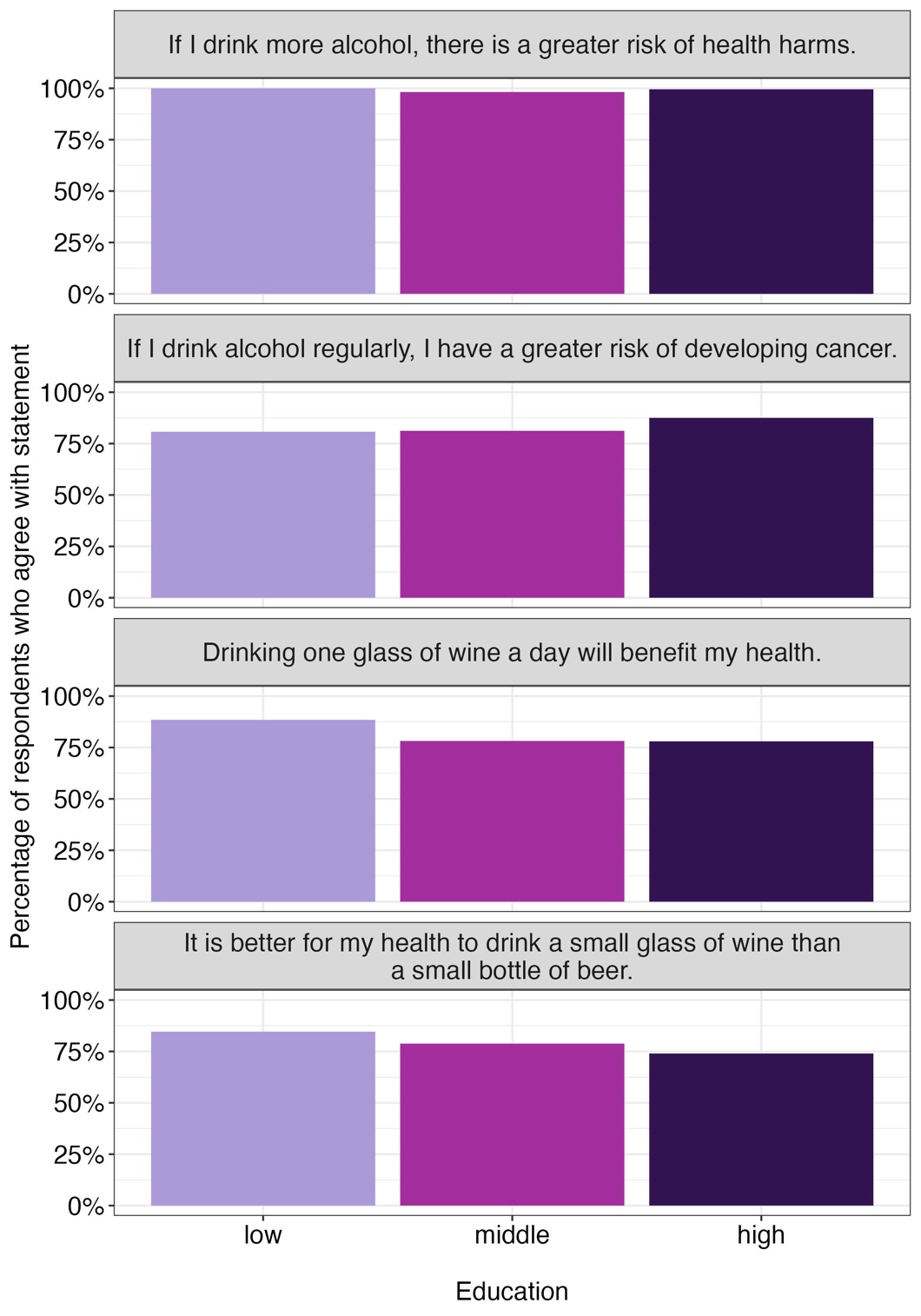


Figure S2. Percentage of respondents who agreed with different statements regarding health harms and benefits of alcohol use, by education group (alcohol health literacy item #3, #4, #5, #6).


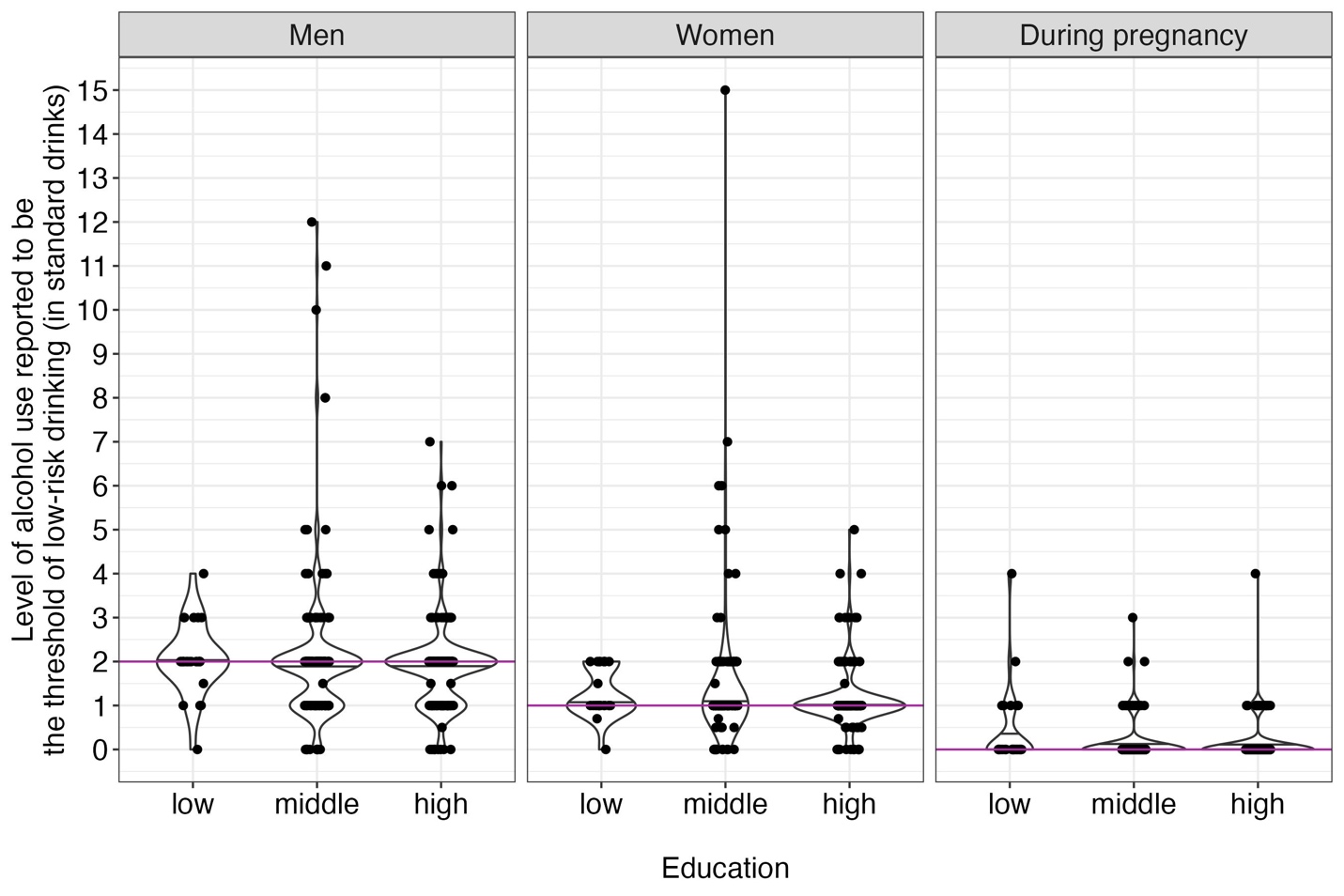


Figure S3. Distribution of the level of alcohol use reported to be the threshold of low-risk alcohol use in standard drinks for men (left panel), women (middle panel), and women during pregnancy (right panel) according to the German alcohol drinking guidelines, by education group (alcohol health literacy item #2, #7, #8). The recommended limits by group are indicated as purple horizontal lines.


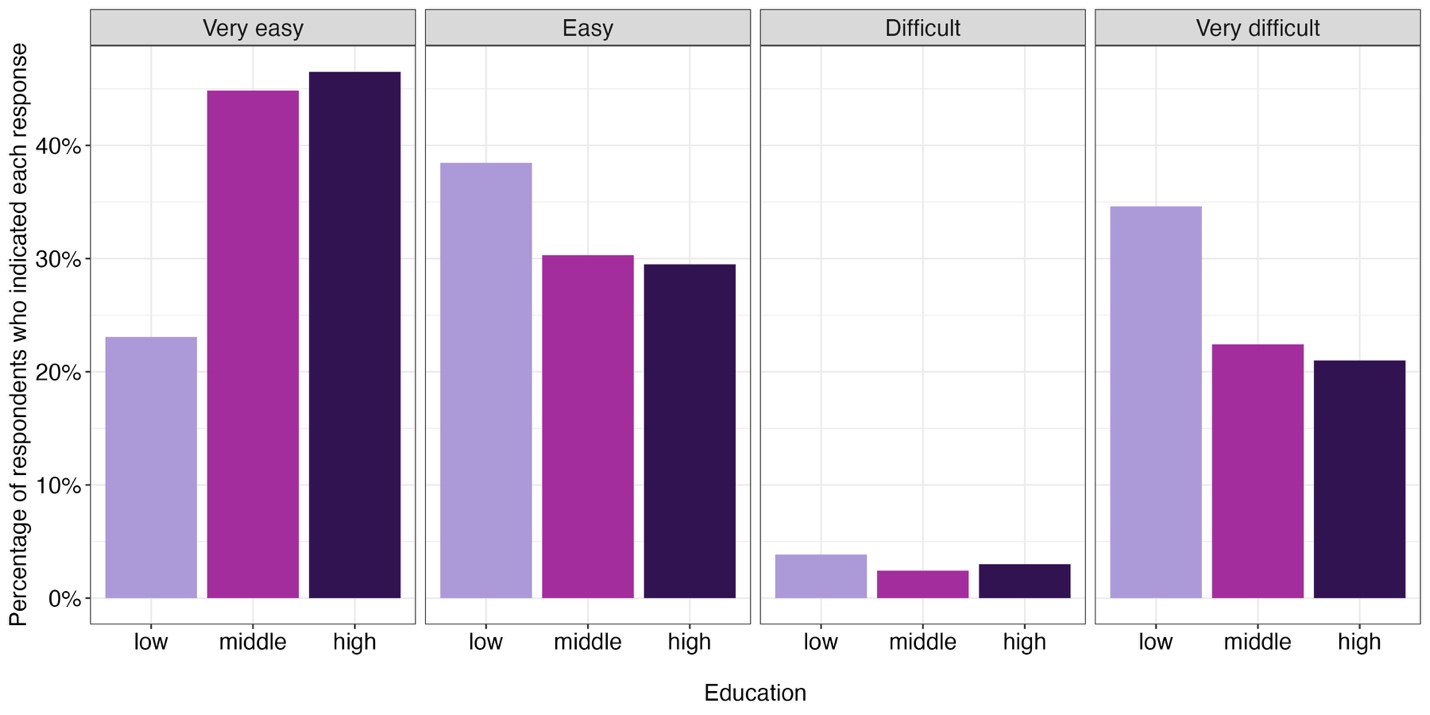


Figure S4. Self-reported ability to understand information on alcohol-related health risks by education group (alcohol health literacy item #9).

Table S2. Multinomial logistic regression models on the association of educational attainment (independent variable) and alcohol health literacy (dependent variable).

|  | **Insufficient AHL** | | | **Missing AHL** | | | |
| --- | --- | --- | --- | --- | --- | --- | --- |
|  | **RR** | **95% CI** | **p-value** | **RR** | | **95% CI** | **p-value** |
| **Model 1** | | | | |  |  |  |
| Educational attainment (ref. high) | | | | |  |  |  |
| Middle | 1.25 | 0.97-1.53 | 0.082 | 1.14 | | 0.78-1.60 | 0.473 |
| Low | **1.53** | **1.06-1.88** | **0.027** | 1.61 | | 0.85-2.44 | 0.130 |
| Gender (ref. men) | | |  |  |  |  |  |
| Women | 0.80 | 0.58-1.08 | 0.156 | **1.38** | | **1.02-1.77** | **0.038** |
| Age groups (ref. 18-34) | | |  |  |  |  |  |
| 35-49 | 1.16 | 0.77-1.59 | 0.453 | 1.15 | | 0.69-1.76 | 0.560 |
| 50+ | 1.14 | 0.84-1.47 | 0.382 | 1.2 | | 0.80-1.71 | 0.368 |
| **Model 2** | | | | | | | |
| Educational attainment (ref. high) | | | | | | | |
| Middle | 1.27 | 0.99-1.56 | 0.057 | 1.17 | | 0.8-1.64 | 0.406 |
| Low | **1.61** | **1.15-1.93** | **0.011** | 1.74 | | 0.94-2.57 | 0.072 |
| Gender (ref. men) | | | | | | | |
| Women | 0.77 | 0.54-1.04 | 0.095 | 1.33 | | 0.97-1.72 | 0.071 |
| Age groups (ref. 18-34) | | | | | | | |
| 35-49 | 1.20 | 0.8-1.65 | 0.35 | 1.19 | | 0.71-1.81 | 0.495 |
| 50+ | 1.19 | 0.88-1.53 | 0.24 | 1.25 | | 0.83-1.77 | 0.276 |
| Alcohol use (ref. low risk) | | | | | | | |
| Non-drinker | **0.44** | **0.23-0.82** | **0.008** | **0.48** | | **0.22-0.98** | **0.043** |
| High risk | 0.88 | 0.64-1.16 | 0.382 | 0.82 | | 0.54-1.21 | 0.333 |
| **Model 3** |  |  |  |  | |  |  |
| Educational attainment (ref. high) | | | | | | | |
| Middle | 1.28 | 1.00-1.57 | 0.052 | 1.16 | | 0.79-1.63 | 0.423 |
| Low | **1.59** | **1.13-1.92** | **0.014** | 1.70 | | 0.91-2.54 | 0.091 |
| Gender (ref. men) | | | | | | | |
| Women | 0.78 | 0.55-1.06 | 0.114 | 1.34 | | 0.98-1.74 | 0.062 |
| Age groups (ref. 18-34), years | | | | | | | |
| 35-49 | 1.20 | 0.80-1.65 | 0.345 | 1.18 | | 0.70-1.80 | 0.511 |
| 50+ | 1.17 | 0.86-1.51 | 0.299 | 1.23 | | 0.81-1.75 | 0.320 |
| Alcohol use (ref. low risk) | | | | | | | |
| Non-drinker | **0.43** | **0.22-0.80** | **0.007** | **0.48** | | **0.22-0.98** | **0.042** |
| High risk | 0.87 | 0.63-1.15 | 0.358 | 0.82 | | 0.54-1.21 | 0.334 |
| General health literacy (ref. sufficient) | | |  |  |  |  |  |
| Problematic | 0.79 | 0.5-1.17 | 0.262 | 1.00 | | 0.56-1.62 | 0.990 |
| Inadequate | 0.99 | 0.6-1.45 | 0.975 | 1.26 | | 0.67-2.04 | 0.436 |

Sample size: n = 508. AHL, alcohol health literacy. CI, confidence interval; RR, risk ratio. Model 1: adjusted for gender and age group. Model 2: adjusted for gender, age group and alcohol use. Model 3: adjusted for gender, age group, alcohol use and general health literacy.
